# MIgGGly (mouse IgG glycosylation analysis) - a high-throughput method for studying Fc-linked IgG N-glycosylation in mice with nanoUPLC-ESI-MS

**DOI:** 10.1038/s41598-018-31844-1

**Published:** 2018-09-12

**Authors:** Olga O. Zaytseva, Bas C. Jansen, Maja Hanić, Mia Mrčela, Genadij Razdorov, Ranko Stojković, Julija Erhardt, Ilija Brizić, Stipan Jonjić, Marija Pezer, Gordan Lauc

**Affiliations:** 1 Genos Glycoscience Research Laboratory, Zagreb, 10000 Croatia; 2 Department of Chemistry, Faculty of Science, University of Zagreb, Zagreb, 10000 Croatia; 3 Division of molecular medicine, Ruđer Bošković Institute, Zagreb, 10000 Croatia; 4 Department of Biology, Faculty of Science, University of Zagreb, Zagreb, 10000 Croatia; 5 Department of Histology and Embryology, Faculty of Medicine, University of Rijeka, Rijeka, 51000 Croatia; 6 Center for Proteomics, Faculty of Medicine, University of Rijeka, Rijeka, 51000 Croatia; 7 Department of Biochemistry and Molecular Biology, Faculty of Pharmacy and Biochemistry, University of Zagreb, Zagreb, 10000 Croatia

## Abstract

Immunoglobulin G (IgG) N**-**glycosylation is crucial for its effector functions. It is a complex trait, and large sample sets are needed to discover multiple genetic factors that underlie it. While in humans such high-throughput studies of IgG N-glycans became usual, only one has been carried out in mice. Here we describe and validate a method for the relative quantification of IgG Fc-linked N**-**glycans in a subclass-specific manner using nano-reverse phase liquid chromatography coupled with mass-spectrometry (nanoRP-LC-MS) applied to murine IgG. High-throughput data processing is ensured by the LaCyTools software. We have shown that IgG isolation procedure is the main source of technical variation in the current protocol. The major glycoforms were quantified reliably with coefficients of variation below 6% for all the analytes with relative abundances above 5%. We have applied our method to a sample set of 3 inbred strains: BALB/c, C57BL/6 and C3H and observed differences in subclass-specific and strain*-*specific N*-*glycosylation of IgG, suggesting a significant genetic component in the regulation of Fc-linked IgG N*-*glycosylation.

## Introduction

Protein glycosylation is an important biological process that refers to the enzymatic attachment of carbohydrates to proteins. Glycosylated proteins (glycoproteins) have been shown to play a role in many biological processes such as cell-cell recognition and immune response^1–4^. The most abundant glycoprotein in plasma of healthy individuals is immunoglobulin G (IgG) that consists of a fragment crystallizable (Fc) and a fragment antibody-binding (Fab) domains. The Fc domain is recognized by various Fcγ receptors and other ligands that mediate its effector functions to direct the immune response. The Fc domain of IgG is highly conserved and contains an N*-*linked glycosylation site in each of its heavy chains^5,6^. The structure of the attached N*-*glycan modulates the affinity of IgG to different types of ligands^7,8^. The absence of core fucose increases IgG affinity for FcγRIIIa leading to enhanced antibody-dependent cell-mediated cytotoxicity (ADCC)^8^, sialylation reduces affinity to FcγRIIIa^9^ and increases affinity for the lectin dendritic cell-specific ICAM3-grabbing non-integrin (DC-SIGN) receptor^7^. Presence of galactose residues on the N-glycan antennae enhances affinity for the complement component C1q^10^.

Glycans can undergo various modifications, such as phosphation of mannose^11^ and sialic acid^3^ residues, sulfation^12,13^ and O-acetylation^14–16^ of sialic acids. O-acetylated sialic acid residues are found in the serum N-glycomes of some vertebrate species such as Atlantic salmon^16^ and rat^14,15^, but are of extremely low abundance in human and mouse serum^14^.

Human and murine IgG can be subdivided into 4 subclasses that differ structurally and functionally. IgG subclasses preferably bind to different Fcγ receptors, possess different abilities to activate the complement system and have specific functions in the immune response^17,18^. Recent studies have shown that different IgG subclasses tend to have distinct Fc region N*-*glycosylation^19,20^, which might contribute to their functionality.

Mouse models are often used to study the immune system and in particular the effector functions of IgG^21–23^. Recently the translation of findings obtained on mouse models into human immunology has been questioned^24,25^. There are profound differences between mouse and human immune systems^26^. Genetic homogeneity and limited exposure to antigens alter the immune response in laboratory animals^24^. Nevertheless, humanized mouse models remain one of the major instruments in the studies of IgG effector functions^27–29^. While these mice express human constant regions of antibodies and Fcγ receptors, their glycosylation machinery is murine. To answer important questions: whether N-glycosylation is responsible for the functional differences between mouse and human immunity and if a particular mouse strain would make a suitable model due to their IgG glycoprofiles, detailed studies of murine IgG N*-*glycosylation are required. A few recent publications described Fc-linked IgG N*-*glycans in common laboratory mouse strains^19,30^ and effects of immunization on antigen*-*specific IgG N**-**glycosylation profile^31^. A large-scale association study of genetic basis of IgG N**-**glycosylation in mice was also conducted^32^. Interestingly, it seems that variability of mouse IgG N*-*glycome is higher than that observed in human populations^32^ and IgG N*-*glycosylation profiles are highly strain-specific^19^, suggesting that proper strain selection is crucial for the studies of IgG effector functions conducted on mouse models.

Commonly used techniques for the measurements of glycans include matrix assisted laser desorption/ionization (MALDI)-time of flight (TOF)-mass spectrometry (MS)^33^, high performance liquid chromatography (HPLC)-fluorescence detection (FD)^19^ and capillary gel electrophoresis with laser-induced fluorescence detection (CGE-LIF)^34,35^. Since a single peak can contain multiple glycan structures^36^, a combination of liquid chromatography (LC) based separation of released glycans and MS-based detection serves as the most informative method for glycoprofiling^37,38^. Recent progress in chemoenzymatic synthesis of glycans made available panels of synthesized N-glycans^39–42^ used as standards for absolute quantifications of N-glycans in complex mixtures^39^. Chemoenzymatically engineered pure preparations of specific IgG glycoforms^40,41,43,44^ are used to study their functional properties. Recently, LC-MS has been used to analyze glycopeptides instead of released glycans. This approach allowed to specifically address Fc-linked glycosylation of IgG in a subclass specific manner. Glycoproteins are enzymatically digested, enriched by hydrophilic interaction liquid chromatography (HILIC) and measured by LC-MS^45,46^. A nanoRP-LC-MS method was developed specifically for the analysis of Fc-linked N*-*glycosylation of human IgG subclasses^47^. Difference in the amino acid sequences of the tryptic peptides results in LC separation of glycoforms based on the amino acid composition of the Fc region of different IgG subclasses. The same approach was used to study Fc N-glycan profiles of four mouse strains^19^. However, no evaluation of the robustness of the method applied to murine samples has been presented.

Studying the genetic regulation of IgG N-glycosylation, its role in immune response and disease requires large sample sizes (>100) due to the small effect sizes and complexity of N-glycan biosynthesis^34^. Therefore, robustness of high-throughput analysis is an important issue. In this study we assessed the reliability of the high-throughput nano-LC-ESI-MS method for quantification of mouse IgG subclass specific Fc N**-**linked glycopeptides. We developed a protocol based on one used for human IgG^47^ and measured the technical variation of the main sample preparation steps and of the LC-ESI-MS analysis of glycopeptides from murine serum samples. We showed that the main source of experimental error comes from IgG isolation on the protein G monolithic plate. Our method allowed quantifying biological variation of N*-*glycosylation traits between the three commonly used laboratory strains. To rule out the effect of age^48^ and sex^20,48^ on IgG N-glycosylation in the cohort we used age- and sex-matched animals raised in the same facility. The main advantage of the current study is that the observed biological variation in the Fc-linked IgG N-glycan profiles can be attributed mainly to the genetic background of the strains. Here we report that the Fc-linked N**-**glycosylation profiles of 15 weeks old male BALB/c, C3H and C57BL/6 mice are strain-specific, which implies a significant genetic component in the regulation of the IgG Fc-linked N**-**glycome.

## Results and Discussion

### Technical variation of sample preparation procedure and analysis

Sample preparation and LC-MS analysis of tryptic glycopeptides could be divided into 5 main steps: 1) IgG isolation on protein G monolithic plate; 2) digestion of the isolated IgG with trypsin; 3) reverse phase solid phase extraction (RP SPE) of the glycopeptides; 4) drying of the glycopeptides; 5) LC-MS analysis and data extraction with LaCyTools software^49^.

**Figure 1:**
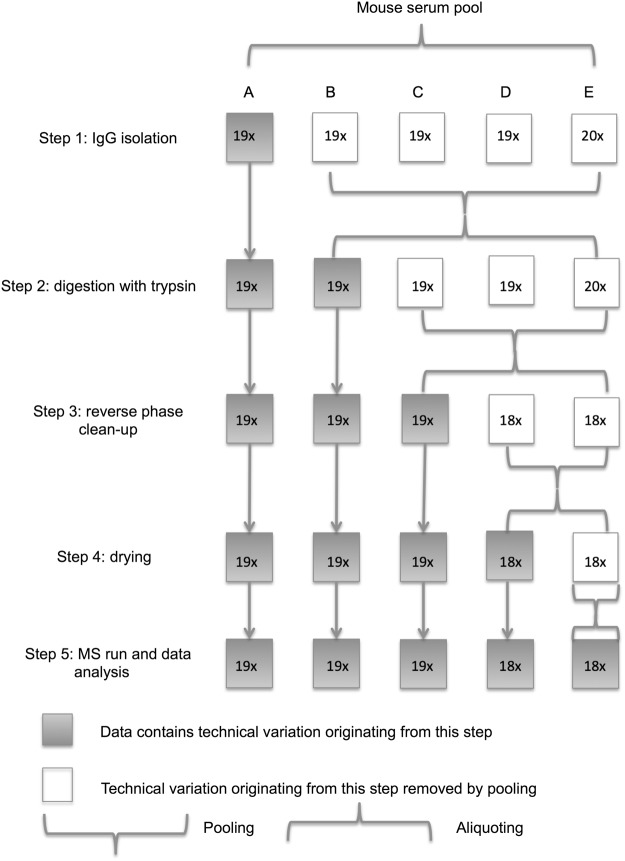
Assessment of the technical variation introduced by the sample preparation procedure and LC-MS analysis. Murine serum pool was aliquoted into the protein G monolithic plate for IgG isolation. The isolated IgG was separated into 5 parts, each corresponding to the conditions A–E, and samples designated for conditions B–E were pooled and aliquoted. Thus, samples A contain technical variation from all sample preparation and analysis steps; samples B contain variation from steps 2–5; C – from steps 3–5; D – from steps 4–5 and samples E contain technical variation resulting only from step 5. The number, preceding the letter “x”, denotes the number of samples per each experimental condition.

To test the technical variation introduced by each step we used aliquots of mouse serum pool. These aliquots were then pooled at different steps of the sample preparation procedure, designated as conditions A–E (18–19 replicates per condition), to get rid of the technical variation introduced by the previous steps (Fig. 1).

In the tryptic digest of mouse IgG 8 glycoforms were quantified by LC-MS for IgG1 and IgG3, 5 for IgG2a/b/c (Supplementary Table S1). In accordance with the previous studies of mouse IgG and plasma N-glycome^14,19,32^, we did not observe any O-acetylated glycoforms in our sample. The distributions of the glycopeptide abundances are shown in Supplementary Fig. 1.

**Figure 2:**
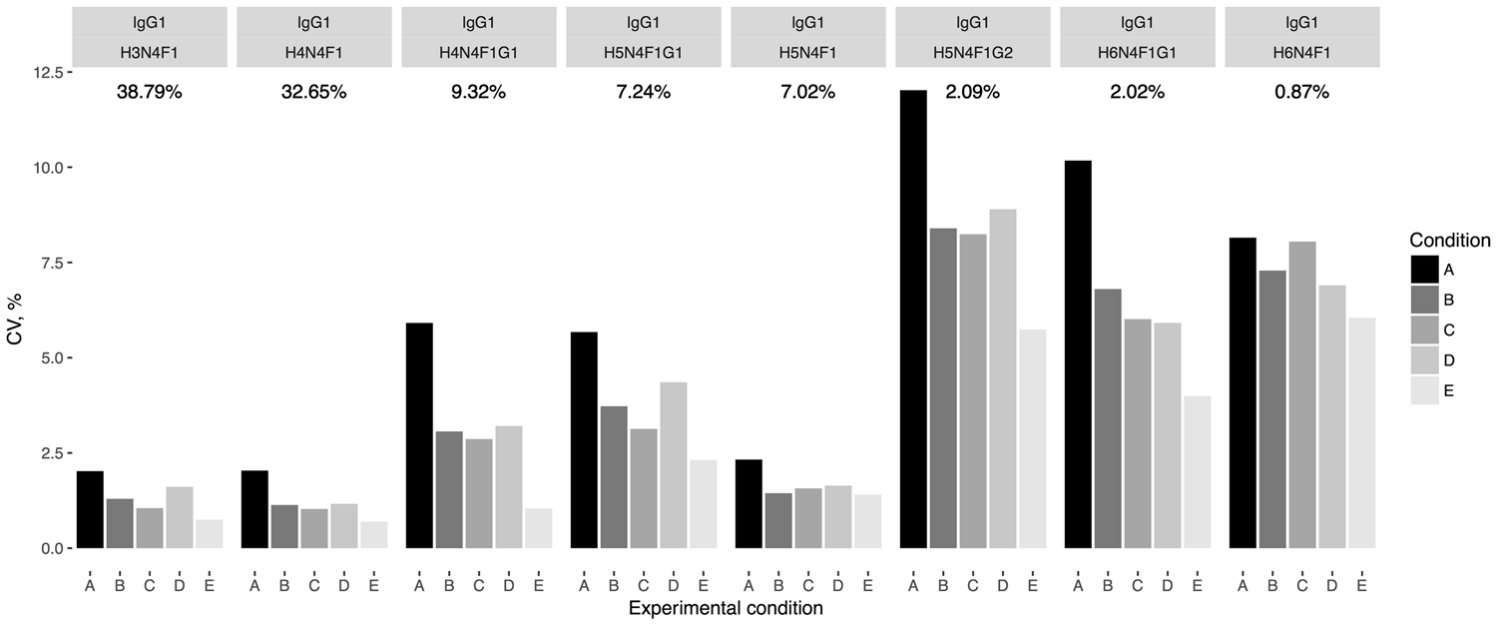
Analysis of variation in the sample preparation and LC-MS analysis for mouse IgG1 glycoforms. Samples A were not pooled; samples B were pooled after IgG isolation; samples C were pooled after tryptic digestion, samples D were pooled after RP-SPE clean-up and samples E were pooled after drying before the LC-MS run. CV – coefficient of variation. Glycan structures names describe the compositions, each letter denotes a sugar residue followed by a number of this type of residues in the N-glycan; H – hexose, N – *N-*acetylglucosamine, F – fucose, G – *N-*glycolylneuraminic acid residues. Under each glycan structure the mean normalized abundance of the glycoform across all 5 conditions is depicted.

Since all the samples represent replicates of the same initial serum pool, all observed experimental variation is purely technical. We showed that most of the variation was introduced by the IgG isolation procedure, while all the subsequent steps did not change much in terms of coefficients of variation (CVs) (Fig. 2, Supplementary Table S1). CVs for condition A representing technical variation introduced by the whole sample preparation and analysis procedures are below 6% for 5 out of 8 IgG1 glycoforms (on average 94.5% of the total integrated area for the subclass), for 5 out of 8 IgG3 glycoforms (96.3% of total integrated area) and for 3 out of 5 IgG2a/b glycoforms (88% of total integrated area). The higher CVs are observed for the least abundant structures (Supplementary Table S1), most of which are sialylated. One possible explanation is that the sialic acids are not stable in the acidic conditions during sample preparation and are known to ionize poorly in positive mode^50^.

As a conclusion, we believe that the major N*-*glycan structures (mean normalized area equal to or above 5%) can be reliably quantified with our method. However, the measurements of the low abundant glycoforms are less accurate and small biological differences between samples might be obscured by the measurement error. To be able to estimate the error introduced by the sample preparation and analysis and to assess the size of the biological effect that can be measured, we recommend including 3–5 standards in the 96-well plate design, especially if the analysis is performed in high-throughput mode.

### Biological variation in 3 commonly used mouse strains

As a working example we have chosen 3 commonly used inbred mouse strains: BALB/c, C57BL/6 and C3H to show the biological variation in subclass-specific Fc-linked N-glycosylation of mouse IgG. All individual mice were 15-week old males kept in the same facility, to exclude environmental confounders such as caging, diet, light regime, etc.

**Figure 3:**
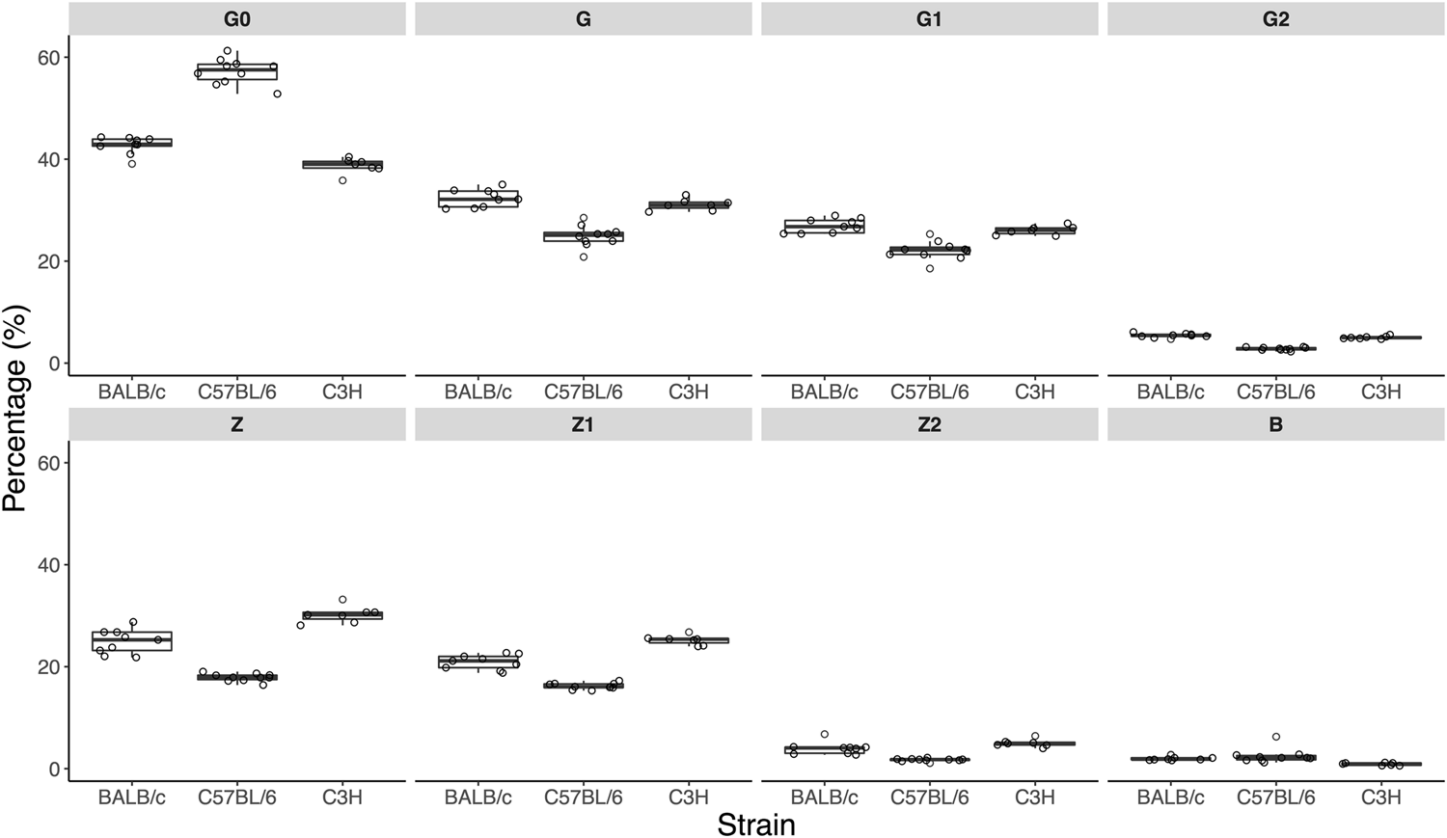
Comparison of Fc-linked IgG1 N*-*glycosylation in the three mouse strains. Derived glycosylation traits (in percentages of relative abundance) are shown as box plots. Each box represents the 1^st^ (Q1) to 3^rd^ (Q3) quartile. Lines inside the boxes represent the median, whiskers extend to the lowest data point within 1.5 *IQR of Q1, and the highest data point within 1.5 *IQR of Q3. Dots represent individual data points. Derived traits represent relative abundance of specific types of N*-*glycan structures in the total IgG1 Fc-linked N*-*glycome: agalactosylated structures, G0; galactosylated structures, G; monogalactosylated structures, G1; digalactosylated structures, G2; sialylated structures, Z; monosialylated structures, Z1; disialylated structures, Z2; structures with bisecting GlcNAc, B.

To compare strains and IgG subclasses in terms of N-glycosylation profiles derived glycan traits were calculated (Supplementary Table S2) that describe abundances of particular types of glycoforms: different degrees of galactosylation, sialylation, structures with bisecting GlcNAc and terminal α1,3-linked galactosylation. Intra-strain variation of the glycoform measurements is shown in Fig. 3 and Supplementary Table S3. Technical variation in this experiment was represented by CVs of the 3 standard serum samples included in the batch (Supplementary Table S3). For all glycoform measurements the technical variation was notably lower than the observed total intra-strain variation, thus the precision of the measurements allowed to detect biological variation of glycoform abundance in the Fc-linked IgG N-glycomes of individual animals.

Even though we designed the experiment trying to minimize the effect of age, sex or facility on IgG glycosylation, for some glycan traits we observed relatively high levels of intra-strain variation (e.g. levels of G0 on Fc-region of IgG3 in all 3 strains). On inter-individual level IgG N-glycosylation could have been affected by the factors we were unable to control: hormonal status of the animals, possible epigenetic effects of embryonic development, lactation, weaning or hormonal status in the parents.

#### Subclass-specific differences in Fc-linked IgG N-glycosylation

The number of glycoforms observed for IgG1, IgG2a/b/c and IgG3 subclasses in all three strains was 7, 8, and 6 respectively (Supplementary Table S4). All subclasses contained glycoforms carrying typical fucosylated biantennary N-glycan structures either with or without terminal galactose and *N-*glycolylneuraminic acid residues. Additionally, structures with terminal α1,3-linked galactose residues were observed in the glycosylation profiles of all three IgG subclasses, although on IgG1 these structures were identified below the quantification limit. Afucosylated structures were either not detected or detected below the quantification limit. IgG1 was the only subclass where a prominent level of structures with bisecting GlcNAc was observed.

Differential N-glycosylation of the Fc-region in IgG subclasses was previously reported both for mice^19^ and humans^20^. In all three strains analysed in this study IgG1 Fc-linked N**-**glycan profile showed the highest abundance of agalactosylated structures and the lowest abundance of galactosylated structures compared to the other subclasses (Supplementary Table S5 and Fig. S2). Interestingly, in this respect N*-*glycan profile of mouse IgG1 is quite similar to that of human IgG4, which also exhibits the highest proportion of agalactosylated fucosylated glycans, low incidence of H5N4F1 and lower incidence of sialylated N-glycans^51^. IgG1 in mice and IgG4 in humans are associated with immunoregulation^21,52^ and both subclasses are induced by type 2 T helper cells^53,54^. In contrast, IgG2 Fc-linked N*-*glycans showed the lowest levels of agalactosylated N-glycans and high abundance of galactosylated glycans and structures with terminal *N-*glycolylneuraminic acid residues (Supplementary Table S5 and Fig. S2). IgG2a/b subclasses in mice could be seen as functionally analogous to human IgG1 or IgG3 subclasses being induced mainly by protein antigens, with high affinity to activating FcγRs^17,55^, IgG2a being particularly effective at inducing ADCC^56^. Previously, lower levels of agalactosylation and higher galactosylation and sialylation were reported for IgG1 and IgG3 in humans as well as for IgG2a/b/c in mice^19,31^. Both murine and human samples show similar glycopatterns for IgG subclasses that are functional counterparts, therefore it is likely that glycoprofiles of particular subclasses reflect their functional role.

#### Strain-specific differences in Fc-linked IgG N-glycosylation

For most of the analysed glycan traits C57BL/6 mice exhibit statistically significant differences from both C3H and BALB/c mice (Supplementary Table S6) after the adjustment of the p-values for 23 tests, while N-glycan profiles of C3H and BALB/c mice are more similar.

As concerns IgG1 glycoforms, C57BL/6 had noticeably lower levels of galactosylated N**-**glycans, more agalactosylated structures and less sialylated N-glycans (Fig. 3, Supplementary Table S3). Levels of agalactosylated N*-*glycan structures with bisecting GlcNAc were similar for BALB/c and C57BL/6 and lowest in C3H strain. It is worth to note that unlike BALB/c and C3H strains, C57BL/6 expresses a rare allotype of IgG1, which among other things is characterized by an amino acid substitution that allowed us to chromatographically separate two IgG1 allotypes and quantify them separately. Sum spectra corresponding to IgG1 glycopeptides found in BALB/c, C3H and C57BL/6 strains are shown in Supplementary Fig. S3.

**Figure 4:**
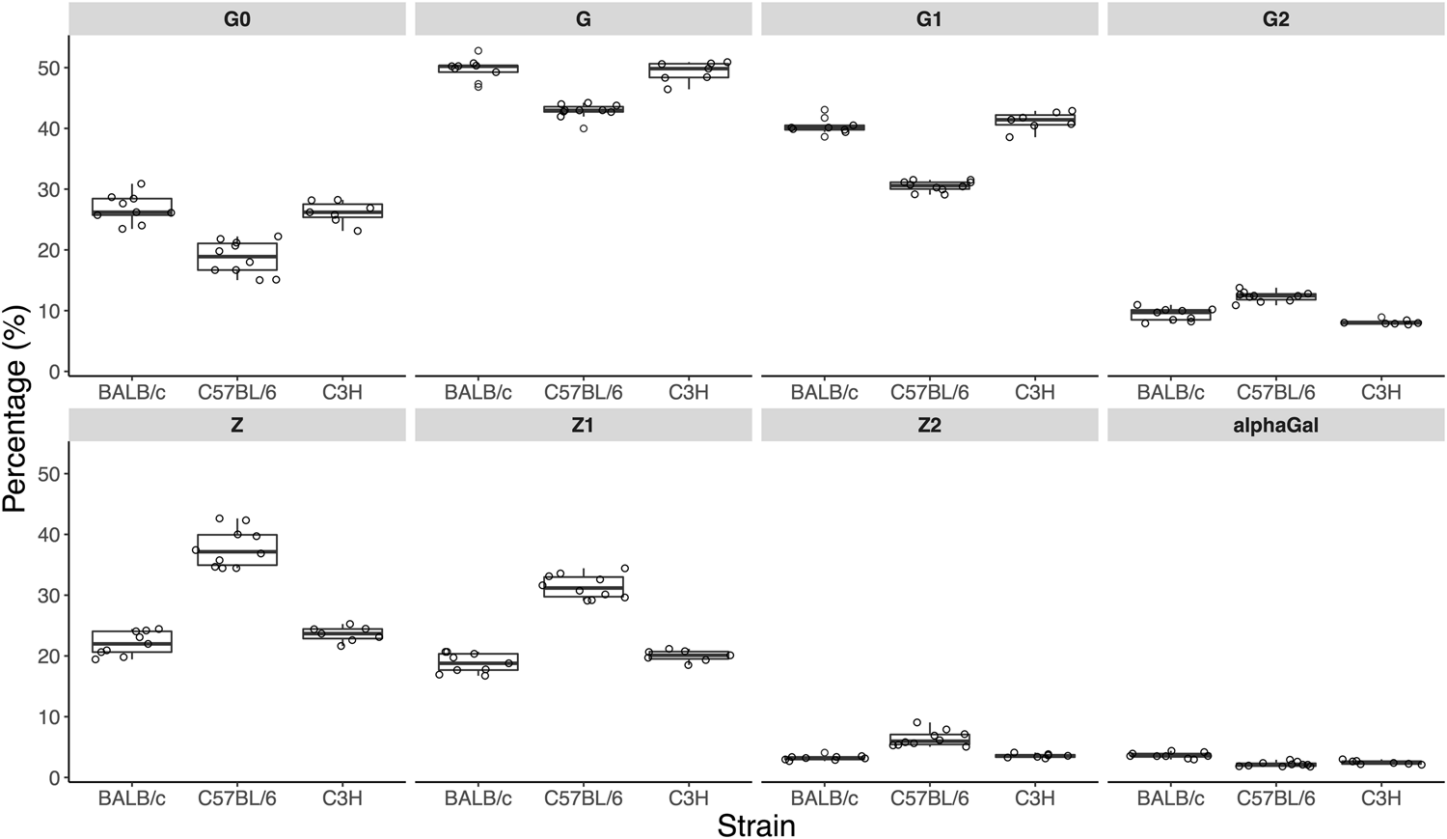
Comparison of Fc-linked IgG2 N-glycosylation in the three mouse strains. Derived glycosylation traits (in percentages of relative abundance) are shown as box plots. Each box represents the 1^st^ (Q1) to 3^rd^ (Q3) quartile. Lines inside the boxes represent the median, whiskers extend to the lowest data point within 1.5 *IQR of Q1, and the highest data point within 1.5 *IQR of Q3. Dots represent individual data points. Derived traits represent relative abundance of specific types of N*-*glycan structures in the total IgG2 Fc-linked N*-*glycome: agalactosylated structures, G0; galactosylated structures, G; monogalactosylated structures, G1; digalactosylated structures, G2; sialylated structures, Z; monosialylated structures, Z1; disialylated structures, Z2.

The N*-*glycan profiles of IgG2a/b/c, however, show a different trend (Fig. 4, Supplementary Table S3). While the incidence of galactosylated N*-*glycans is lower in C57BL/6 animals, agalactosylation levels in this strain are also the lowest and sialylation is the highest among the three strains analysed, almost twice as high as in BALB/c and C3H mice. Incidence of structures with α1,3**-**linked galactose is the highest in BALB/c with 3.6% of those structures in IgG2 N-glycome and similar in C3H and C57BL/6.

**Figure 5:**
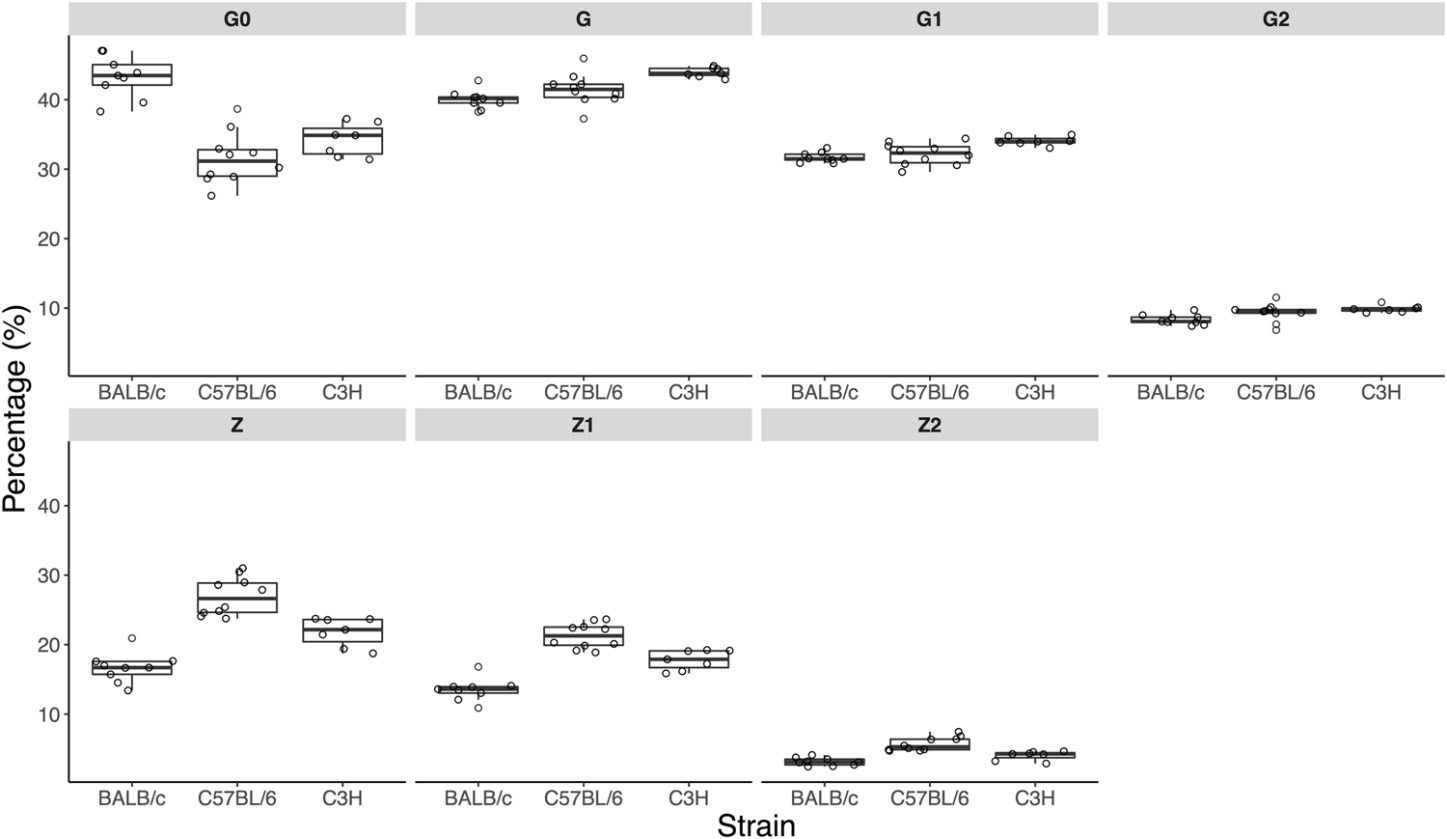
Comparison of Fc-linked IgG3 N*-*glycosylation in the three mouse strains. Derived glycosylation traits (in percentages of relative abundance) are shown as box plots. Each box represents the 1^st^ (Q1) to 3^rd^ (Q3) quartile. Lines inside the boxes represent the median, whiskers extend to the lowest data point within 1.5 *IQR of Q1, and the highest data point within 1.5 *IQR of Q3. Dots represent individual data points. Derived traits represent relative abundance of specific types of N*-*glycan structures in the total IgG3 Fc-linked N*-*glycome: agalactosylated structures, G0; galactosylated structures, G; monogalactosylated structures, G1; digalactosylated structures, G2; sialylated structures, Z; monosialylated structures, Z1; disialylated structures, Z2.

The highest abundance of agalactosylated structures on IgG3 is observed in BALB/c mice compared to C57BL/6 and C3H mice (Fig. 5, Supplementary Tables S3, S6). Galactosylation is the highest in C3H and the lowest in BALB/c, while incidence of N-glycans with terminal sialylation is the highest in C57BL/6 and lowest in BALB/c mice.

The observed differences in the N-glycan traits could have various biological causes and/or consequences. Low levels of sialylated N-glycans might be connected with increased risk of autoimmune diseasess^27,57^. However, the biological impact of differences in the Fc-linked N-glycome compositions has to be evaluated in context of abundances of different IgG subclasses in the serum. For instance, in C57BL/6 mice the proportion of sialylated glycans is the highest out of the three strains on the “activating” subclasses IgG2a/b and the lowest on the “protective” subclass IgG1. It is possible that glycosylation on the “activating” subclasses in this strain acts as protection from inflammation. A question remains whether the Fc-linked N-glycome composition at steady state could be predictive of the pathway immune response takes upon antigen challenge. To answer it further research of IgG N-glycome changes during immune response in different mouse strains is needed.

The experiment was designed to exclude the confounding factors of age, sex and environmental differences. The Fc-linked N-glycan profiles of different IgG subclasses were still found to be predominantly strain*-*specific, which agrees with the previous studies^19,32^. This implies that the observed differences are largely defined by the differences of genetic and/or epigenetic backgrounds characteristic for the analysed strains. It is unlikely that the differences in the N*-*glycan profiles are rooted only in the general expression and/or activity of the enzymes involved in N*-*glycan biosynthesis, *e.g*. the level of sialylation in C57BL/6 is the lowest of the three strains on IgG1, but the highest on IgG3. This suggests subclass-specific regulation of Fc-linked N*-*glycosylation and may be explained by the different biological roles of the IgG subclasses. The whole immunological context, including antigen dose, immunization route, antigen presentation and cytokine milieu, might play a role in directing glycosylation of IgG subclasses in the same way as it directs class switching to a particular IgG subclass in IgG producing B cells^58–60^.

The genome wide association studies of IgG N*-*glycosylation traits indicate that IgG N-glycosylation is not controlled solely by the genes encoding glycosyltransferases^34,61^. Mutations in genes coding for transcription factors and chromatin remodelling proteins, in particularly those involved in class-switch and transcriptional activity control in B-cells, like IKZF1 and BACH2^61^ and even genes encoding constant region of immunoglobulin heavy chains in mice might influence IgG glycosylation^32^. The latter makes pronounced differences between the two murine allotypes of IgG1 especially interesting (consistent with^19^). In conclusion, careful selection of the murine strain with characteristic IgG N-glycoprofile that best matches the experimental hypothesis is essential, as the strain genetic background can have a significant impact on the observed results.

## Methods

### Chemicals, reagents and consumables

Protein G monolithic plates were acquired from BIA separations (Ajdovščina, Slovenia). AcroPrep Advance 0.45 μm hydrophilic polypropylene (GHP) filter plates were obtained from the Pall Corporation (Ann Arbor, MI, USA). ThermoFischer Scientific provided 0.2 ml skirted 96-well robotic plates and the Acclaim PepMap100 C8 (5 mm × 300 μm i.d.) trap column (Waltham, MA, USA). The Halo C18 nano-LC column (150 mm × 75 μm i.d., 2.7 μm HALO fused core particles) was supplied by Advanced Materials Technology (Wilmington, DE, USA). Chromabond C_18_ec beads were obtained from Marcherey-Nagel (Düren, Germany). Modified trypsin of sequencing grade was purchased from Worthington Biochemical Corporation (Worthington, MI, USA). Phosphate-buffered saline (PBS) was prepared in house from sodium chloride (for analysis, Gram-Mol, Zagreb, Croatia), potassium chloride (for analysis, Kemika, Zagreb, Croatia), sodium phosphate, dibasic, 99+% and potassium phosphate, monobasic, 99+%, ACS reagent (Acros Organics, ThermoFischer Scientific). Formic acid (FA) 98–100% of EMSURE ACS, Reag. Ph Eur purity and ammonium bicarbonate of BioUltra, >=99.5% purity were acquired from Merck (Darmstadt, Germany). TFA of HPLC purity was supplied by Sigma-Aldrich (Darmstadt, Germany). ACN of LC-MS purity was obtained from JT Baker (ThermoFischer Scientific).

### Samples

Serum pools of BALB/c and C3H mice were obtained from the Medical Faculty of Rijeka University, Rijeka, Croatia and the Department of Biology, Faculty of Science, University of Zagreb, Croatia, respectively. Serum samples of 26 individual male mice aged 15 weeks of strains BALB/c, C3H and C57BL/6 (9, 10 and 7 individuals per strain respectively) were obtained from Institute Ruđer Bošković, Zagreb, Croatia. All the experiments were carried out with the appropriate ethics approvals from the Croatian Ministry of Agriculture in accordance with relevant guidelines and regulations.

### IgG isolation from serum, tryptic digestion and RP SPE purification

Immunoglobulin G was isolated from mouse serum using protein G monolithic plates as described previously^62^. The dried eluates containing purified IgG were resuspended in 400 µL ultrapure water. Approximately 10–20 µg IgG was transferred into a 0.2 mL skirted 96-well Robotic Plate and digested with 200 ng trypsin overnight at 37 °C. The resulting glycopeptides were purified by reverse phase solid phase extraction (RP SPE) using C_18_ beads as described in^63^, dried by vacuum centrifugation and dissolved in 20 μL ultrapure water.

### LC-MS analysis of IgG tryptic glycopeptides

Tryptic digests were analysed on a nanoACQUITY UPLC system (Waters, USA) coupled to a Compact MS (Bruker Daltonics, Bremen, Germany) as described in^63^. In brief, 9 μL eluates containing IgG tryptic glycopeptides was loaded on a trap column and washed for 1 min with 0.1% TFA (mobile phase A) at a flow rate of 40 μL/min. Separation was achieved on a C_18_ nano-LC column using a 3.5 min gradient at a flow rate of 1 μL/min from 18.5% to 25% solvent B (80% ACN). Spectra were recorded in positive mode. The method is based on the original method described in^47^.

### Data processing

Glycopeptide compositions were assigned based on *m/z* value (Supplementary Table S7) and isotopic pattern. Peak areas were integrated with LaCyTools v 1.0.1 b.7 software and normalized to the total integrated area per IgG subclass^49^. LaCyTools first identified the observed retention time t_r_(o) for 4 user-defined glycopeptide clusters, by identifying the peak maximum of an extracted ion chromatogram (±0.1 Th.) within a given t_r_ range (±30 s) around the expected retention time t_r_(e). The t_r_ calibration was done by performing a power law function through at least 4 of the t_r_(o) and t_r_(e) pairs where the t_r_(o) peak had a S/N ratio greater than 9. Subsequently, the t_r_ calibrated measurements were used to retrieve all MS^1^ spectra belonging to the 4 user-defined glycopeptide clusters. Sum spectra of each glycopeptide cluster were then created by binning the spectra (n = 33 per second), and *m/z* calibrated using a similar approach as the t_r_ calibration. Briefly, the accurate *m/z* of a calibrant peak was determined as the maximum of a univariate spline fit through the raw data points. An accurate and exact m/z pair would only be used for calibration if the S/N of the accurate *m/z* was greater than 9. The calibration was then performed on a minimum of 4 accurate-exact *m/z* pairs, by applying a 2^nd^ degree polynomial through them. A list of user-specified glycopeptides in [M + 2 H]^2+^ and [M + 3 H]^3+^ was quantified using the calibrated sum spectra. LaCyTools integrated individual isotopologues of each glycopeptide until at least 95% of the total theoretical isotopic distribution was quantified. The glycopeptide area was then adjusted by subtracting the background area, *i.e*. the region with the lowest average intensity within ±10 Th of the monoisotopic peak. Furthermore, LaCyTools used the area of the individual isotopologues to calculate the isotopic pattern quality (IPQ), which together with the S/N ratio and mass accuracy was used to verify the peak identity. Peaks with S/N below 9, IPQ above 40% and m/z values after calibration noticeably deviating from the theoretical ones were not integrated.

### Analysis of the source of variation

Aliquots of 100 μL of the serum pool (mixture of serum of BALB/c and C3H mice) were loaded onto the protein G monolithic plate for IgG isolation. During further sample preparation 18–19 replicates were pooled at different steps of the procedure to remove the technical variation, introduced by the preceding steps (Fig. 1): A – not pooled at any step; B – after step IgG isolation; C – after tryptic digestion; D – after clean up on C18 beads; E – after drying in a vacuum concentrator before MS analysis and subsequent data processing.

### Statistical analysis and data visualisation

For each glycopeptide abundances of [M + 2 H]^2+^ and [M + 3 H]^3+^ charge states obtained with LaCyTools were summed. To make measurements across samples comparable, normalization by total area was performed: the integrated area of each glycopeptide was divided by the total integrated area of glycopeptides corresponding to respective IgG subclass within a sample. Derived traits representing relative abundance of specific types of N-glycan structures in the total Fc-linked N-glycome of each subclass were calculated (Supplementary Table S2). Prior to statistical analysis all measurements were log-transformed due to the right skewness of their distributions. Analyses of associations between strain and glycan traits were performed using a one-way ANOVA testing, followed up by post-hoc tests for the glycan traits with uncorrected ANOVA p**-**values < 0.05. P-values obtained in Tukey post-hoc tests were adjusted for 23 hypotheses tested by controlling false discovery rate with the Benjamini-Hochberg procedure.

Data was analyzed and visualized using R programming language (version 3.3.2). Contributing authors created all the figures.

## Electronic supplementary material

Supplementary Information

Dataset 1

Dataset 2

Dataset 3

Dataset 4

Dataset 5

Dataset 6

Dataset 7

## Data Availability

The data generated in the current study are available in form of the mzXML files containing LC-MS measurements from the Mendeley Data repository^64–66^.
