# Supplementary material for: MIgGGly (mouse IgG glycosylation analysis) - a high-throughput method for studying Fc-linked IgG N-glycosylation in mice with nanoUPLC-ESI-MS
Source: Sci Rep. 2018 Sep 12;8:13688. doi: 10.1038/s41598-018-31844-1 (PMC6135756; doi:10.1038/s41598-018-31844-1)
Supplement: Supplementary file 1 — Supplementary Information [file 41598_2018_31844_MOESM1_ESM.docx]

**MIgGGly (mouse IgG glycosylation analysis) - a high‑throughput method for studying Fc-linked IgG N‑glycosylation in mice with nanoUPLC-ESI-MS**

**Olga O. Zaytseva^1,*,+^, Bas C. Jansen^1,+^, Maja Hanić^1^, Mia Mrčela^2^, Genadij Razdorov^1^, Ranko Stojković^3^, Julija Erhardt^4^, Ilija Brizić^5,6^, Stipan Jonjić^5,6^, Marija Pezer^1^, and Gordan Lauc^1,7^**

**Supplementary Figure 1.** **Distributions of glycan measurements in the experimental conditions A-E.** Samples A were not pooled; samples B were pooled after IgG isolation; samples C were pooled after tryptic digestion, samples D were pooled after RP-SPE clean-up and samples E were pooled after drying before the LC-MS run. Glycan structures names describe the compositions, each letter denotes a sugar residue followed by a number of this type of residues in the N-glycan; H – hexose, N – *N-*acetylglucosamine, F – fucose, G – *N-*glycolyl-neuraminic acid residues. N-glycoform measurements (in percentages of relative abundance) are shown as box plots. Each box represents the 1^st^ (Q1) to 3^rd^ (Q3) quartile. Lines inside the boxes represent the median, whiskers extend to the lowest data point within 1.5 * IQR of Q1, and the highest data point within 1.5 *IQR of Q3. Dots outside of the boxes represent outliers and big dots inside the boxes represent the mean.

 **Supplementary Figure S2.** **Comparison of Fc-linked N-glycosylation in the four mouse IgG subclasses: IgG1 (IgG1F + IgG1I), IgG2 (IgG2a + IgG2b), IgG3 in BALB/c, C3H and C57BL/6 mice.** Derived glycosylation traits (in percentages of relative abundance) are shown as box plots. Each box represents the 1st (Q1) to 3rd (Q3) quartile. Lines inside the boxes represent the median, whiskers extend to the lowest data point within 1.5 * IQR of Q1, and the highest data point within 1.5 *IQR of Q3. Dots represent the outliers (data points with values lower than Q1 - 1.5*IQR or higher than Q3 + 1.5*IQR). Derived traits represent relative abundance of specific types of N-glycan structures in the total Fc-linked N-glycome of a corresponding subclass: agalactosylated structures, G0; neutral galactosylated structures, G; neutral monogalactosylated structures, G1; neutral digalactosylated structures, G2; sialylated structures, Z; monosialylated structures, Z1; disialylated structures, Z2.

**Supplementary Figure S3. Examples of sum spectra of IgG1 glycopeptides.** Each panel shows a sum spectrum obtained from an individual sample of BALB/c, C3H and C57BL/6 mice. Peaks corresponding to [M+2H]^2+^ and [M+3H]^3+^ glycoforms are denoted with respective glycan structures.
